# The Potential Role of *Gymnema inodorum* Leaf Extract Treatment in Hematological Parameters in Mice Infected with *Plasmodium berghei*

**DOI:** 10.1155/2021/9989862

**Published:** 2021-06-28

**Authors:** Sakaewan Ounjaijean, Suriyan Sukati, Voravuth Somsak, Orawan Sarakul

**Affiliations:** ^1^School of Health Sciences Research, Research Institute for Health Sciences, Chiang Mai University, Chiang Mai 50200, Thailand; ^2^School of Allied Health Sciences, Walailak University, Nakhon Si Thammarat 80161, Thailand; ^3^Research Excellence Center for Innovation and Health Products, Walailak University, Nakhon Si Thammarat 80161, Thailand

## Abstract

Malaria remains a significant cause of death in tropical and subtropical regions by serious complications with hematological abnormalities consistent with high parasitemia. Hence, this study aimed to determine the efficacy of the *Gymnema inodorum* leaf extract (GIE) on hematological alteration in *Plasmodium berghei* infection in mice. Groups of ICR mice were infected intraperitoneally with parasitized red blood cells of *P. berghei* ANKA (PbANKA). They were administered orally by gavage of 100, 250, and 500 mg/kg of GIE for 4 consecutive days. Healthy and untreated groups were given distilled water, while 10 mg/kg of chloroquine was treated as the positive control. Hematological parameters including RBC count, hemoglobin (Hb), hematocrit (Hct), mean corpuscular volume (MCV), mean cell hemoglobin (MCH), mean cell hemoglobin concentration (MCHC), RBC distribution width (RDW), white blood cell (WBC) count, and WBC differential count were measured. The results showed that significant decreases of RBC count, Hb, Hct, MCV, MCH, MCHC, and reticulocytes were observed in the untreated group, while RDW was significantly increased compared with the healthy control. Furthermore, the WBC, neutrophil, monocyte, basophil, and eosinophil of untreated mice increased significantly, while the lymphocyte was significantly decreased compared with the healthy control. Interestingly, GIE normalized the hematological alteration induced by PbANKA infection in GIE-treated groups compared with healthy and untreated groups. The highest efficacy of GIE was observed at a dose of 500 mg/kg. Our results confirmed that GIE presented the potential role in the treatment of hematological alteration during malaria infection.

## 1. Introduction

Malaria remains a public health burden caused by a parasite in the genus *Plasmodium* and transmitted by the female *Anopheles* mosquito. Five species of *Plasmodium* are infectious to humans, including *Plasmodium falciparum*, *Plasmodium vivax*, *Plasmodium malariae*, *Plasmodium ovale*, and *Plasmodium knowlesi* [1]. It is estimated that 90% of malaria-related deaths occur in sub-Saharan Africa, most of which are children under five years old. The death due to malaria parasite infection is caused by critical complications such as cerebral malaria, severe hemolytic anemia, metabolic acidosis, respiratory distress, liver dysfunction, acute kidney injury, and hematological abnormalities [2]. Malaria-induced hematological abnormalities are also associated with hemoglobinopathy, nutritional status, and immunity [3, 4]. However, several research studies revealed the increase in development and spread of antimalarial resistance by malaria parasites and toxicities associated with standard antimalarial drugs [5]. Hence, the search for alternatives, especially from plants that present an antimalarial and a wide range of biological activities, is urgently needed.

*Gymnema inodorum* (Lour.) Decne. (Phak Chiang Da as a local name) belongs to the Asclepiadaceae family and has been known to be effective for diseases, including diabetes mellitus, gout, and rheumatoid arthritis. This plant is indigenous in Southeast Asia including Thailand, particularly in the northern part of the country, and is widely consumed [6]. *G. inodorum* leaf extract showed high antioxidant and anti-inflammatory activities with polyphenols and flavonoids as the major compounds [7]. It has also been found that the leaf extract of *G. inodorum* could decrease blood glucose by inhibiting glucose absorption [8, 9]. *G. sylvestre* is the same genus that is popular in India for suppressing glucose absorption and preventing type 2 diabetes mellitus [10, 11]. The leaves of this plant also have polyphenols, flavonoids, triterpenoid saponins, anthraquinones, alkaloids, tannin, quinones, and gymnemic acids, which are the major active compounds of *G. sylvestre* leaf extract [12]. *In vitro* and *in vivo* studies of *G. sylvestre* leaf extract described the pharmacological properties, including antidiabetic, anticancer, antibacterial, antioxidant, anti-inflammatory, antimalarial, hepatoprotective, and immunosuppressive activities [13]. In addition, gymnemic acids and other constituents have been found in *G. inodorum* leaf extract, so this plant might have properties found in *G. sylvestre*. However, the studies regarding the hematological effect of *G. inodorum* in the rodent malaria model have never been carried. In malarial studies using rodent models, *P. berghei* is a suitable model due to the most common characteristics with *P. falciparum* [14]. Therefore, it is necessary to evaluate *G. inodorum* leaf extract's therapeutic potential on hematological abnormalities during *P. berghei* infection in mice.

## 2. Materials and Methods

### 2.1. Chemicals

Chloroquine diphosphate salt (CQ) purchased from Sigma (Sigma Chemical, St Louis, MO, USA) was used in the present study. All chemicals and reagents were of analytical grade and procured from the certificated suppliers.

### 2.2. Collection of Plant Material

The leaves of *Gymnema inodorum* was obtained from the Chiangda organic company garden (Chiang Mai, Thailand). This plant was identified correctly and authenticated by the plant biologists at Chiang Mai University. The voucher specimen of the plant was deposited in the Research Institute for Health Sciences, Chiang Mai University, Chiang Mai, Thailand.

### 2.3. Preparation of the Aqueous Leaf Extract of *Gymnema inodorum*

Fresh leaves of *G. inodorum* were washed with clean water and dried at 60°C in a hot air oven overnight. The dried leaves were ground into powder in a mortar with pestle and subsequently pulverized into fine powder using an electric blender. The powdered plant material was extracted in distilled water (DW) at a proportion of 5 g%. This mixture was allowed to incubate at 60°C in the incubator shaker at 300 rpm for 30 min. Centrifugation was carried out at 2,500 rpm for 15 min, and then the supernatant was collected. After filtration through Whatman no. 1 filter paper, the filtrate was lyophilized to dryness. The *G. inodorum* leaf extract (GIE) was stored at −20°C before experiment [15].

### 2.4. Preparation of the Antimalarial Drug

CQ at a dose of 10 mg/kg was freshly prepared corresponding to the mouse's body weight in 0.3 ml of DW and treated orally by gavage.

### 2.5. Animal Preparation

The experimental mice used in the present study were male ICR mice, aged about 4–6 weeks, weighing 25–30 g, and purchased from the Nomura Siam International Co., Ltd. They were housed in the animal room with temperature control (25 + 0.5°C) and 12 h light-12 h dark cycle. The mice were fed with the standard pellet diet (CP082) and clean drinking water, *ad libitum*. The experiments involving animals were carried out following the guideline for the care and use of laboratory animals and were approved by the animal care and use committee, Walailak University (WU-AICUC-63-031).

### 2.6. *Plasmodium berghei*

The rodent malaria parasite *Plasmodium berghei* strain ANKA (PbANKA) was obtained from the MR4 (Malaria Research and Reference Reagent Resource Center). The stock of parasitized red blood cells (RBC) of PbANKA was inoculated intraperitoneally into the donor naïve ICR mice. Parasitemia was monitored daily by microscopy of Giemsa-stained blood film. The mice parasitized with PbANKA recording 20–30% parasitemia were then sacrificed, and blood was collected by cardiac puncture. Blood was diluted in phosphate buffer solution (PBS) to obtain 1 × 107 parasitized RBC in the volume of 0.3 ml, and the naïve ICR mice were infected.

### 2.7. Parasitemia Determination

Blood was collected from the tail vein of each mouse and used to make a thin blood smear. The smear was fixed with absolute methanol and subsequently stained with 10% Giemsa for 10 min. Percent parasitemia was determined by counting the number of parasitized RBC out of 2,000 RBC in random fields under a microscope with a 100x oil immersion lens. Percent parasitemia was calculated according to the following formula:

(1) $$\begin{array}{c} \text{\% parasitemia}=\frac{\text{total number of parasitized RBC}\times 100}{\text{total number of RBC}}. \end{array}$$

### 2.8. Hematological Parameter Determination

Mice were sacrificed, and blood was collected by cardiac puncture into the EDTA vacuum tubes. Complete blood count (CBC) including RBC count, hemoglobin (Hb), hematocrit (Hct), mean corpuscular volume (MCV), mean cell hemoglobin (MCH), mean cell hemoglobin concentration (MCHC), RBC distribution width (RDW), reticulocytes, white blood cell (WBC) count, and WBC differential was subsequently performed using the automate analyzer system at the Research Institute for Health Sciences, Walailak University. In addition, EDTA blood was also diluted with 1% brilliant cresyl blue in an equal volume. After mixing gently, incubation at 37°C for 15 min was performed. The smear was subsequently prepared, and reticulocytes were counted under a light microscope using a 100x oil immersion lens. Percent reticulocytes were calculated according to the following formula:

(2) $$\begin{array}{c} \text{\% reticulocyte}=\frac{\text{total number of reticulocyte RBC}\times 100}{\text{total number of RBC}}. \end{array}$$

### 2.9. Effect of GIE on Hematological Parameters in PbANKA-Infected Mice

Standard Peters' test was carried out to determine the effect of GIE on hematological parameters in mice infected with PbANKA [14]. Naïve ICR mice were divided into 6 groups of 3 mice each. Group I was neither infected with PbANKA nor treated with the extract as healthy control. Group II was infected with PbANKA but was not treated with the extract as the untreated control. Group III, IV, and V were infected with PbANKA and treated with 100, 250, and 500 mg/kg of GIE, respectively. Group VI was infected with PbANKA and treated with 10 mg/kg of CQ as a positive control. The treatment was administered orally by gavage once a day for 4 consecutive days (day 0–3). On day 4, the mice were sacrificed, and blood from each mouse was collected by cardiac puncture. Fresh blood was used to prepare a thin blood smear for parasitemia determination and the measurement of CBC using an automated analyzer.

### 2.10. Data Analysis

All statistical analyses were performed using the GraphPad Prism software (GraphPad Prism software, Inc., USA). The values were expressed as mean + standard error of mean (SEM), and comparisons were made using one-way ANOVA with Tukey's post hoc test. Statistical significance was considered at 95% confidence, *p* < 0.05.

## 3. Results

### 3.1. Hematological Alteration of RBC in GIE-Treated Mice

**Figure 1:**
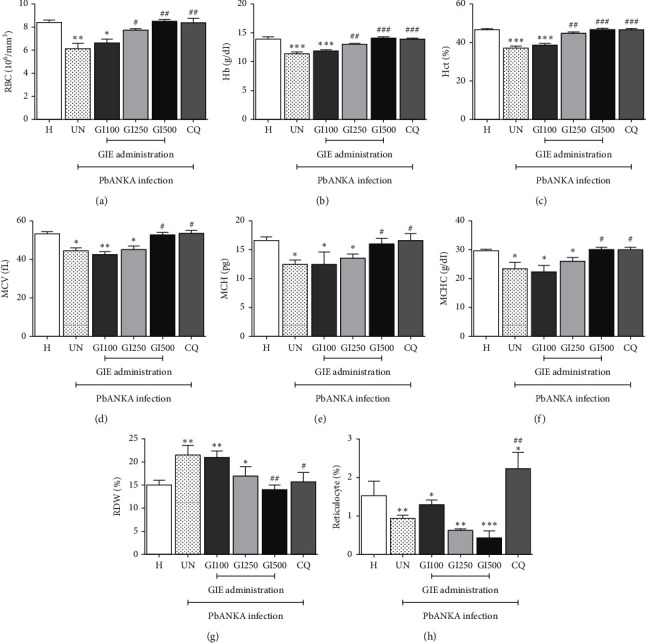
Effect of GIE on hematological alteration of RBC in PbANKA-infected mice. Groups of mice were infected intraperitoneally with 1 × 10^7^ parasitized RBC of PbANKA and treated orally with 100, 250, and 500 mg/kg of GIE for 4 consecutive days. Hematological parameters of RBC were measured: (a) RBC count, (b) Hb, (c) Hct, (d) MCV, (e) MCH, (f) MCHC, (g) RDW, and (h) reticulocyte. H: healthy mice; UN: untreated mice; GI100, GI250, and GI500: 100, 250, and 500 mg/kg of GIE-treated mice, respectively; CQ: 10 mg/kg of CQ-treated mice. ^*∗*^*p* < 0.05, ^*∗∗*^*p* < 0.01, and ^*∗∗∗*^*p* < 0.001, compared with H. ^#^*p* < 0.05, ^##^*p* < 0.01, and ^###^*p* < 0.001, compared with UN. Results were presented as mean + SEM.

The results of hematological parameters of RBC are presented in Figure 1. PbANKA-infected mice in the untreated group induced significant (*p* < 0.05) decreases in RBC count, Hb, Hct, MCV, MCH, MCHC, and reticulocytes, when compared with the healthy control (Figures 1(a)–1(f) and 1(h), UN). RDW was found to be significantly (*p* < 0.01) higher in the untreated mice than the healthy control. The treatment of infected mice with GIE at a dose of 500 mg/kg and CQ restored the altered hematological indices compared with the healthy control. There were significant (*p* < 0.05) differences when the hematological indices of the GIE- and CQ-treated groups were compared with those of the untreated mice (Figures 1(a)–1(h), GI500 and CQ). This indicated that the GIE normalized the hematological alteration of RBC generated by PbANKA infection. However, reticulocytes were significantly (*p* < 0.05) decreased in GIE-treated groups and increased for the CQ-treated group when compared with the healthy control (Figure 1(h), GI100, GI250, GI500, and CQ).

### 3.2. Hematological Alteration of WBC in GIE-Treated Mice

**Figure 2:**
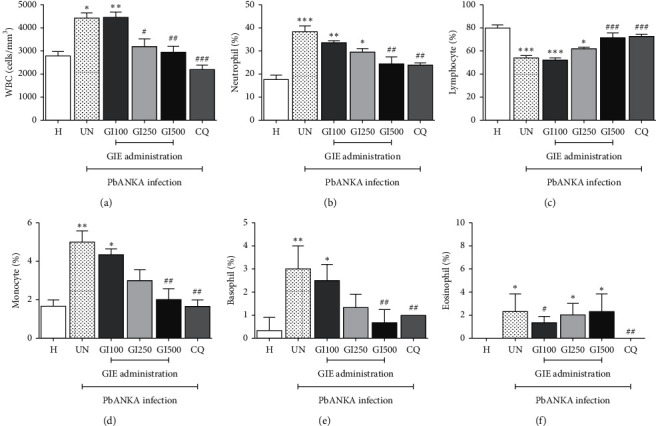
Effect of GIE on hematological alteration of WBC in PbANKA-infected mice. Groups of mice were infected intraperitoneally with 1 × 10^7^ parasitized RBC of PbANKA and treated orally with 100, 250, and 500 mg/kg of GIE for 4 consecutive days. Hematological parameters of WBC were measured: (a) WBC count, (b) neutrophil, (c) lymphocyte, (d) monocyte, (e) basophil, and (f) eosinophil. H: healthy mice; UN: untreated mice; GI100, GI250, and GI500: 100, 250, and 500 mg/kg of GIE-treated mice, respectively, CQ: 10 mg/kg of CQ-treated mice. ^*∗*^*p* < 0.05, ^*∗∗*^*p* < 0.01, and ^*∗∗∗*^*p* < 0.001, compared with H. ^#^*p* < 0.05, ^##^*p* < 0.01, and ^###^*p* < 0.001, compared with UN. Results were presented as mean + SEM.

The alteration of hematological parameters of WBC in PbANKA-infected mice treated with GIE is presented in Figure 2. There was a significantly (*p* < 0.05) elevated WBC, neutrophil, monocyte, basophil, and eosinophil count, while a significant (*p* < 0.001) decrease in lymphocytes was observed in untreated groups compared with the healthy control (Figures 2(a)–2(f), UN). The GIE at a dose of 500 mg/kg and CQ normalized the hematological alteration of WBC during PbANKA infection in mice (Figures 2(a)–2(e), GI500 and CQ). This implied that GIE restored WBC homeostasis in mice infected with PbANKA. However, eosinophils were significantly (*p* < 0.05) higher in infected mice treated with GIE compared with the healthy control (Figure 2(f), GI100, GI250, and GI500).

## 4. Discussion

In this study, GIE showed the ability to restore hematological parameters induced by PbANKA infection in mice. Hematological parameters of RBC and WBC are common biomarkers of malaria infection and indicators of compounds against malaria infection. The results showed that significant decreases in RBC count, Hb, Hct, MCV, MCH, MCHC, and reticulocytes as well as increased RDW were observed in the untreated group compared with healthy control. This suggested that infected mice suffered from severe hemolysis and had hypochromic microcytic anemia during PbANKA infection [3, 16]. It might be due to rapid RBC destruction and lysis of both parasitized and uninfected RBC, bone marrow and erythropoietic suppression, and dyserythropoiesis [17, 18]. In addition, it has also been reported that an increase in RBC fragility led to hemolysis followed by anemia in the PbANKA-infected mice [19]. Several studies have revealed that proinflammatory cytokines such as interferon-*γ* (IFN-*γ*), tumor necrosis factor-*α* (TNF-*α*), macrophage migration inhibitory factor, and hemozoin played an important role in the pathogenesis of hemolysis and anemia during malaria infection by erythropoiesis resistance, decreasing reticulocytosis, and inhibiting erythropoietin-induced erythroid precursor proliferation [20–23]. Moreover, malaria infection increased immunoglobulin-G autoantibody against uninfected RBC, decreasing RBC deformability and improving erythrophagocytosis [24]. According to our previous observation that GIE had no antimalarial activity (data not shown), the ability of GIE to restore RBC count, Hb, Hct, MCV, MCH, MCHC, and RDW as well as significantly decrease reticulocytes when compared with the healthy and untreated groups suggested that the GIE might process the protective effect and immunomodulating and erythropoietic activities. These effects would be due to the presence of active metabolites in the GIE. The effect of GIE on hematological parameters of RBC was consistent with the standard antimalarial drug CQ. It was found that CQ-treated mice showed a significant increase in reticulocytes when compared with the healthy and untreated groups. It has been reported that CQ could result in increased erythropoietin levels in association with its anti-inflammation, resulting from an increase in reticulocytes [25]. However, it appeared that the activity of the lower doses of GIE (100 and 250 mg/kg) was not strong enough to protect hemolysis and anemia during PbANKA infection in mice. This might be due to the low levels of active compounds in the extract whose activity could not be detected.

WBC plays a role in the immune defense against foreign antigens through the processes of leukocytosis and antibody production. Significantly increased WBC count, neutrophils, and monocytes and decreased lymphocytes in PbANKA-infected mice without treatment were observed. These results suggested that stimulation of the immune system by infection might be developed as a physiological response [26]. During malaria infection, phagocytosis activity by neutrophils and monocytes was first responded, while lymphocytes were still decreased [27]. Lymphocytes have an important role in the immune system to produce antibodies against malaria parasites after the phagocytic activity occurred [28]. Moreover, basophils and eosinophils were significantly increased in the untreated group, responsible for mediating inflammatory and cytotoxic events associated with malaria infection. Basophils play a crucial role in inflammatory reactions recruited to the sites of inflammation and drive proinflammatory responses [29, 30]. The present study, the ability of GIE to normalize the hematological parameters of WBC in the GIE-treated groups, especially at a dose of 500 mg/kg, showed that the extract had immunomodulatory, antioxidant, and anti-inflammatory effects. These activities have been linked to the presence of polyphenols, flavonoids, alkaloids, terpenoids, tannin, and other phenolic compounds of the extracts consistent with GIE, which also contains these compounds [31]. Additionally, gymnemic acids, a major active compound presented in the GIE, might be considered to exert the protective effect on the alteration of hematological parameters induced by PbANKA infection in mice [32]. However, the active compounds responsible for the results in this study need to be further identified.

## 5. Conclusion

This study demonstrated that GIE has the potential effect on significantly protecting hematological alteration during PbANKA infection in mice. This effect might be attributed to active constituents in this extract, which may have acted singly or in synergy with others to show the activity observed in this study. However, the identification and characterization of the active compounds in the extract are warranted. Additionally, the active compounds should also be studied in clinical trials. Therefore, it justifies the use of GIE in the development of alternative agents for the management of malaria.

## Data Availability

The data used to support the findings of this study are available at https://figshare.com/s/912935cb2e7d5370b478 (DOI: 10.6084/m9.figshare.14298872).
